# CENP-A nucleosome—a chromatin-embedded pedestal for the centromere: lessons learned from structural biology

**DOI:** 10.1042/EBC20190074

**Published:** 2020-07-28

**Authors:** Ahmad Ali-Ahmad, Nikolina Sekulić

**Affiliations:** 1 Centre for Molecular Medicine Norway (NCMM), Nordic EMBL Partnership, Faculty of Medicine, University of Oslo, Oslo 0318, Norway; 2 Department of Chemistry, University of Oslo, P.O. Box 1033, Blindern NO-0315, Norway

**Keywords:** CENP-A, centromere, chromatin

## Abstract

The centromere is a chromosome locus that directs equal segregation of chromosomes during cell division. A nucleosome containing the histone H3 variant CENP-A epigenetically defines the centromere. Here, we summarize findings from recent structural biology studies, including several CryoEM structures, that contributed to elucidate specific features of the CENP-A nucleosome and molecular determinants of its interactions with CENP-C and CENP-N, the only two centromere proteins that directly bind to it. Based on those findings, we propose a role of the CENP-A nucleosome in the organization of centromeric chromatin beyond binding centromeric proteins.

## Introduction

During cell division, centromeres play a fundamental role in directing the accurate segregation of sister chromatids. They mediate the recruitment of multiprotein megastructures, ‘kinetochores’. Kinetochores attach to microtubule­based spindle fibers that subsequently pull condensed chromosomes to opposite poles of the dividing cell [1]. Centromere defects have been reported to result in aneuploidy, cell death, and cancer [2]. The centromeric locus has specific DNA and protein composition. In complex eukaryotes, centromeric DNA is usually repetitive (termed as α­-satellites in humans). Bioinformatics analysis has shown that centromeric DNA evolves very fast and shows little conservation between species [3,4]. Furthermore, the discovery of neocentromeres [5–7], new functional centromeres formed at ectopic loci (often as a consequence of the disruption of the original centromere), has reinforced the idea that a specific DNA sequence is not essential for centromere function. In contrast, the protein component of the centromere (in complex eukaryotes called **c**onstitutive **c**entromere **a**ssociated **n**etwork (of proteins) - CCAN) is a hallmark of all functional centromeres, although its composition varies between species [8]. Interestingly, only one of the proteins in this complex, CENP-B, recognizes a particular DNA sequence (17-bp CENP-B box) [9,10] but its presence is neither conserved nor necessary for centromere function [5,8,11]. This information led to the conclusion that centromeres are defined epigenetically. In particular, numerous experiments have helped to establish the histone H3 variant CENP-A as an epigenetic mark of the centromere. CENP-A is sufficient to seed and propagate a functional centromere/kinetochore [12–20].

What the specific features of CENP-A are, and how they help to establish and maintain the centromere, have been critical questions in the field for the last couple of decades. Intensive work on the reconstitution of chromatin and centromeric complexes from purified components, together with advances in cryo-electron microscopy, have paved the way toward understanding the molecular determinants of centromeric chromatin. Here, we review the atomic structures of the CENP-A nucleosome (in isolation and in complex with other centromeric proteins) obtained to date, and we speculate on how the presence of CENP-A nucleosomes can modify chromatin architecture.

## Structure of the CENP-A nucleosome

The presence of the centromere-specific histone H3 variant CENP-A is a conserved feature in most eukaryotic centromeres [21], although its sequence is changing fast across species [22–25]. Rapid evolution of CENP-A is connected with co-evolution of other centromere and kinetochore proteins [8,21] and with the evolution of underlining DNA [26]. Thus, although mediating the essential and evolutionally conserved process of chromosome segregation, the centromeres have a different architecture and compositions that are species specific. In this review, we will focus mainly on human and yeast CENP-A nucleosome analysis as most of the structural work has been done with proteins from these species.

**Figure 1:**
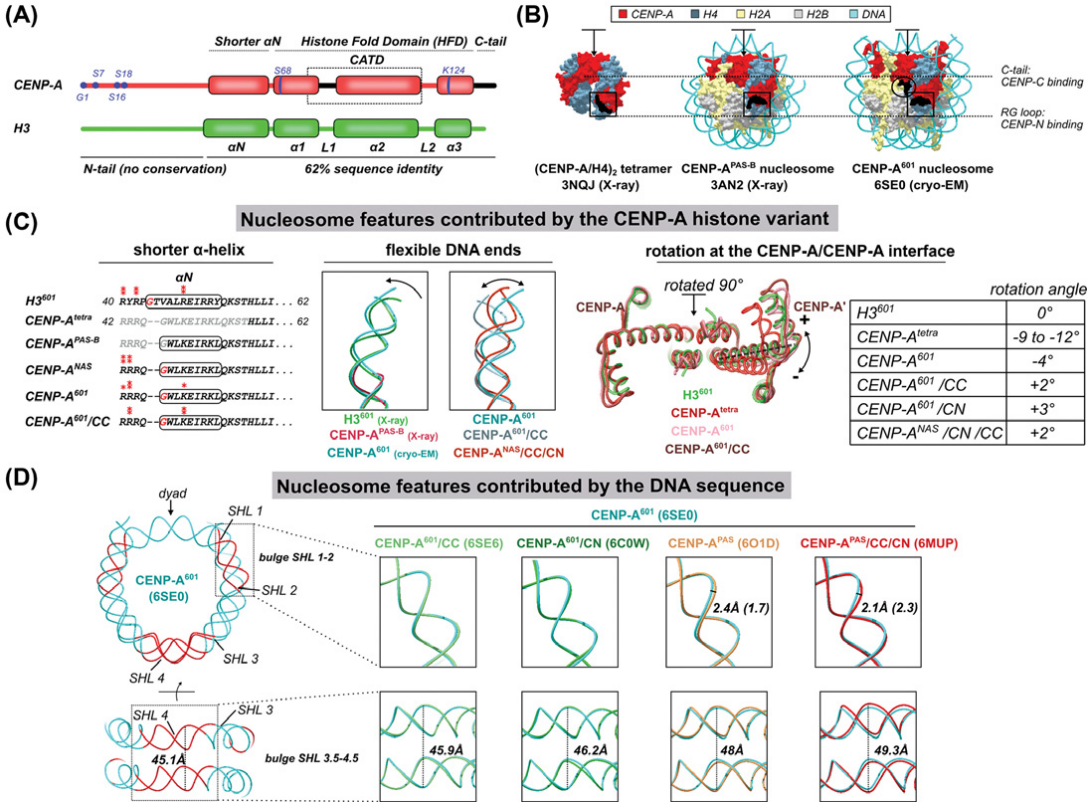
Structural features of CENP-A nucleosome (**A**) Schematic representation of H3 and CENP-A. The post-translational sites on CENP-A are colored in blue and the L1 loop and C-terminal tail, which specifically bind CENP-N and CENP-C respectively, are colored in black. (**B**) Surface representation of CENP-A-containing (sub)nucleosomal complexes, solved by X-ray crystallography and cryo-EM. CENP-A is in red, H4 is in blue, H2A is in yellow, H2B is in gray and the DNA is in cyan. CENP-A-specific features recognized by CENP-C (C-terminal tail) and CENP-N (RG-loop) are shown in black and boxed on each of the structures. The arrow is showing the position of the four-helix bundle between two CENP-A molecules (rotated 90° in (**C**), right). PDB ID and the experimental method used are indicated below each structure. (C) Nucleosome features contributed by CENP-A. Left: Multiple sequence alignment, illustrating a shorter α-N helix (boxed sequence) of CENP-A, observed in various structures. Residues interacting with DNA are labeled with one star if only one side of the nucleosome is involved in interactions, and with two stars if residues on both sides are interacting. Middle: overlay of terminal DNA in H3 nucleosome (3LZ0) and CENP-A nucleosome structures obtained by X-ray crystallography (3AN2) or cryo-EM (6SE0). The flexibility of terminal DNA is modulated by the binding of CCAN components (6SE0, 6MUP, 6SEE). Right: Rotation at the four-helix bundle formed between two CENP-A or two H3 molecules (indicated with the arrow in (B) and rotated 90° away from the viewer). The angle between α2 helices (amino acids 86–113) in the canonical nucleosome is taken as a reference. The rotation is most pronounced in the (CENP-A/H4)_2_ tetramer (3NQJ) and is greatly reduced in the CENP-A nucleosome (6SE0), while it virtually disappears when CENP-C and CENP-N bind the CENP-A nucleosome (6MUP). A table summarizing the measured rotation angles is shown on the right. (**D**) Nucleosome features contributed by the DNA sequence. Left: the DNA of the CENP-A^601^ nucleosome (6SE0) is shown in cyan and regions where important DNA path deviation is observed, in comparison with nucleosome wrapped in the α-satellite DNA, are highlighted in red. The distance between DNA gyres is measured between I-36 phosphate and I-41 phosphate. In order to highlight differences in the DNA path around the nucleosome in budge SHL 1–2 and budge SHL 3.5–4.5, on the right, are shown as zoomed views of different overlays. The first four panels show that there are no significant differences in the DNA path between the CENP-A^601^ nucleosome (cyan; 6SE0) and the CENP-A^601^ nucleosome, in complex with CENP-C (light green, 6SE6), or with CENP-N (dark green, 6C0W). The overlay of the CENP-A^601^ nucleosome (cyan; 6SE0) with the CENP-A^PAS^ nucleosome (orange; 6O1D) reveals a 2.4-Å deviation in the SHL 1–2 (1.7 Å at the opposite side of the nucleosome) and 2 Å widening of DNA gyres. When the CENP-A^601^ nucleosome (cyan; 6SE0) is overlaid with the CENP-A^PAS^ nucleosome with two CENP-C and two CENP-N molecules (orange; 6O1D), the difference at the SHL 1–2 is 2.4 Å (2.3 Å on the other side of the nucleosome) and widening between DNA gyres increases to 49.3 Å. All nucleosomes were overlaid by aligning the histone cores. Abbreviation: CENP-A^601^, CENP-A nucleosome on super-positioning 601 sequence. CENP-A^PAS^, CENP-A nucleosome on palindromic α-satellite DNA sequence.

CENP-A is the most divergent histone H3 variant in human (Figure 1A) [27]. In its histone-fold domain (HFD) and associated αN helix, it shares 62% sequence identity with the canonical H3, while the histone tails are highly dissimilar between the two. As a consequence, human CENP-A is subject to a unique, and limited, set of post-translational modifications (reviewed in [28]) (Figure 1A). Post-translational modifications are implicated in the CENP-A deposition at centromeres, its stability and maintenance, recruitment of CCAN proteins and in the specific organization of centromeric chromatin [28].

The indispensable role of CENP-A in epigenetic specification of centromeres and the relatively high sequence divergence, compared with other H3 variants, have long been motivators for understanding its structure within the chromatin. Over the years, studies done with proteins from different organisms, using both natural and artificial DNA sequences and employing different biophysical techniques, have led to various models for the CENP-A containing nucleosomes, evoking a different histone stoichiometry and composition along with a different DNA path (reviewed in [29]). However, to date all atomic resolution structures of CENP-A nucleosomes (Table 1) are octameric in nature with negatively supercoiled DNA, highly resembling canonical nucleosomes. Rather than altering the composition or the overall shape of the nucleosome, CENP-A is inferring unique dynamic properties to the otherwise expected nucleosome shape.

**Table 1:** High-resolution structures of CENP-A (sub)nucleosome

PDB	DNA	Species	Method	Stabilizing reagents	CCANs	Res. (Å)	Reference
**CENP-A subnucleosomal complex: CENP-A/H4 tetramer**							
3NQJ	-	*Homo sapiens*	X-ray	-	-	2.1	[31]
3NQU	-	*Homo sapiens*	X-ray	-	-	2.5	[31]
**CENP-A nucleosome**							
3AN2	Palindromic α-Satellite with CENP-B boxes (PAS-B)	*Homo sapiens*	X-ray	-	-	3.6	[35]
6O1D	α-Satellite (NAS)	*Homo sapiens*	Cryo-EM	-	-	3.4	[40]
6E0P	α-Satellite (NAS)	*Homo sapiens*	Cryo-EM	scFv*	-	2.6	
6E0C	601	*Homo sapiens*	Cryo-EM	scFv*	-	2.6	
6SE0	601	*Homo sapiens*	Cryo-EM	-	-	3.8	[37]
6UPH	601	*Saccharomyces cerevisiae*	Cryo-EM	Tween 20	-	2.7	[44]
6TEM	601	*Homo sapiens* and *Xenopus laevis*	Cryo-EM	-	-	3.9	[41]
**CENP-A/H3.3 hybrid nucleosome**							
3WTP	Palindromic α-Satellite (PAS)	*Homo sapiens*	X-ray	-	-	2.7	[45]
**CENP-A nucleosome in complex with CCAN component(s)**							
6BUZ	601	*Homo sapiens*	Cryo-EM	-	1x CENP-N	3.9	[88]
6C0W	601	*Homo sapiens*	Cryo-EM	-	1x CENP-N	4	[89]
6MUP	α-Satellite (NAS)	*Homo sapiens*	Cryo-EM	Glutaraldehyde	2x CENP-C 2x CENP-N	3.5	[48]
6MUO	α-Satellite (NAS)	*Homo sapiens*	Cryo-EM		2x CENP-C 1x CENP-N	3.6	
6SE6	601	*Homo sapiens*	Cryo-EM	-	2x CENP-C	3.5	[37]
6SEG	601	*Homo sapiens*	Cryo-EM	-	2x CENP-C	3.1	
6QLD	601	*Saccharomyces cerevisiae*	Cryo-EM	BS3†	CCAN	4.1	[46]
**H3-CENP-A-H3 tri-nucleosome**							
6L49	601	*Homo sapiens*	Cryo-EM	Mg^2+^	-	18.9	[43]

Abbreviations: NAS, natural (non-palindromic) α-satellite DNA sequence; PAS, palindromic (engineered) α-satellite DNA sequence; PAS-B, PAS that has CENP-B box at the ends.

* scFv - engineered a single-chain fragment from the PL2-6 antibody, which includes the variable heavy and light chains connected by a flexible linker.

† BS3, bis(sulfosuccinimidyl)suberate.

In solution, CENP-A forms stable dimers with histone H4 that further dimerizes to form dimer-of-dimers or (CENP-A/H4)_2_ tetramers (similar to the canonical (H3/H4)_2_ tetramer). Hydrogen-deuterium exchange studies of the (CENP-A/H4)_2_ tetramer, indicated special solution dynamics imposed by CENP-A [30], inferring a more compact and rigid complex compared with the canonical counterpart. The first atomic resolution X-ray crystal structure of the human (CENP-A/H4)_2_ tetramer [31] clearly identified stronger hydrophobic interactions at the CENP-A/H4 interface, versus the H3/H4 interface and 9–12° rotation at the dimer/dimer interface, providing an explanation for solution studies (Figure 1B,C). The region conferring CENP-A rigidity in solution was mapped to loop 1 (L1) and the α2 helix within HFD and named CENP-A targeting domain (CATD) for its ability to drive centromeric localization when swapped in the H3 histone sequence. Later studies [32–34] showed that CATD is being recognized by the specific CENP-A chaperone HJURP, which ensures deposition at the centromere. The first high-resolution structure of the CENP-containing nucleosome came from crystallographic studies on human nucleosomes [35] (Figure 1B). The structure revealed an H3-like nucleosome with two copies of H2A, H2B, H4 and CENP-A and negatively supercoiled DNA, albeit disordered at the ends. Despite this, the high structural similarity with the canonical nucleosome could have been enforced by crystal contacts. Several high-resolution cryo-EM structures of the CENP-A nucleosome were recently published, which largely confirmed the findings observed in the X-ray structure. The cryo-EM structures also revealed fine details contributed by species-specific histone variants, DNA sequence and the binding of other proteins to the CENP-A nucleosome. Here, we summarize what we have so far learned about the unique features of the CENP-A nucleosome, contributed by the histone variant itself and structural features emphasized by α-satellite DNA.

### Nucleosome features contributed by histone variant CENP-A

#### Shorter αN-helix and flexible DNA ends

The human CENP-A, just like H3, has a disordered N-terminal tail followed by an αN helix and a typical histone fold, consisting of three α-helices (Figure 1A). While the helices in the histone fold are stable, the αN helix of CENP-A is dynamic. In the absence of DNA, in (CENP-A/H4)_2_ tetramer, this helix is completely disordered [30,31] (Figure 1C, left). When the CENP-A packs in the nucleosome, the DNA stabilizes the αN helix, but to a lesser extent than the analogous helix of H3 in the canonical nucleosome. The αN helix in CENP-A is half a turn shorter than in H3 due to the position of the amino acids ^46^GW^47^ in CENP-A, which interfere with helix formation (glycine is a helix breaker) (Figure 1C). Furthermore, αN helix in H3 interacts with DNA through bi-dentate H3^R49^ interaction, which is replaced by the weaker CENP-A^K49^. The shorter αN helix, together with looser DNA binding, contribute to increased dynamics of the DNA ends in CENP-A nucleosome, clearly observed in solution during MNase digestion [31,35–38]. Shorter CENP-A nucleosomal DNA is in agreement with CENP-A ChIP-seq data, indicating that this feature is retained in cellular context [39]. The flexible DNA is observed in almost all structures of the CENP-A nucleosome, independently of the species, DNA sequence, or methods used [35,37,40–44]. Indeed, in the heterotypic human nucleosome with one copy of H3 and one copy of CENP-A [45], the flexibility of the DNA is clearly associated with the CENP-A part of the nucleosome, while the DNA on the H3 side is stably wrapped. The most extreme flexibility of the DNA ends in human nucleosomes are observed in the X-ray structure of the human CENP-A nucleosome [35] where the terminal 13-bp pairs on each DNA end are disordered. The use of an engineered palindromic DNA sequence with a CENP-B binding motif at the ends, and tight packaging in the crystal, might have contributed to pronounced DNA dynamics. Interestingly, the cryo-EM structure of the budding yeast CENP-A nucleosome assembled on the super-positioning 601 sequence, reveals almost the same extent of DNA disorder [44], while the binding of CCAN [46] further unwraps DNA, leaving only 105 bp to interact with the histone core. In contrast, the cryo-EM structures of the human CENP-A nucleosome in isolation [37,40] show traceable density for almost the entire 145–147 bp sequence of the nucleosomal DNA, albeit further from the nucleosome core than in the case of H3 nucleosomes, and with less intensity [47] indicating some degree of flexibility. A recent cryo-EM study on CENP-A nucleosome [41] exploited phase plate technology to decipher differential 601 DNA unwrapping from the two sides of the CENP-A nucleosome. However, while the nucleosomal DNA sequence generally had a minor effect on the terminal DNA flexibility in the reported CENP-A nucleosome in solution [37], the binding of the CCAN proteins showed an impact [37,48]. The binding of CENP-C potentiates DNA dynamics, indirectly through the destabilization of the C-terminal tail of H2A [37], but upon subsequent CENP-N binding, DNA tails become more rigid [48] (Figure 1C, middle). Although the structure of the CENP-A nucleosome in complex with part of CENP-C and CENP-N [48] demonstrates that both proteins can concomitantly bind the nucleosome, some studies are suggesting a dynamic nature of these associations through the cell cycle [49–51]. Thus, it is possible that the extent of terminal DNA dynamics on the CENP-A nucleosome also changes through the cell cycle as a consequence of modulations by different binding partners. One might also wonder what would be the functional implication of altered nucleosome DNA dynamics. Studies on nucleosomes assembled on longer DNA [42,43] show that the flexible nature of the DNA at the entry/exit sites results in an alternative path of the linker DNA, which does not cross above the dyad and thus cannot accommodate the binding of the histone H1. In this context, flexible DNA ends on the CENP-A nucleosome would translate into a unique chromatin structure specific for the centromeric region. Additionally, flexible DNA ends might help accommodate binding of other CCAN components, that was impaired when the CENP-A nucleosome was deprived of its terminal DNA flexibility [42,46]. In summary, the shorter αN helix and the looser DNA binding are intrinsically well-conserved features of the CENP-A nucleosome that are not heavily influenced by the DNA sequence itself, but are modulated by CCAN binding.

#### Rotation at the CENP-A/CENP-A interface

Another highly unique part of the CENP-A nucleosome is the tetramerization interface between two CENP-A/H4 dimers (Figure 1C). This interface is mediated exclusively with the CENP-A residues which make a four-helix bundle just below the dyad (Figure 1B, arrow). SAXS experiments in solution showed the (CENP-A/H4)_2_ tetramer to be much more compact than the (H3/H4)_2_ tetramer [31] and the crystal structure of the (CENP-A/H4)_2_ tetramer shows substantial (9–12°) rotation at the dimer–dimer interface. This rotation is significantly reduced when CENP-A forms nucleosomes (Figure 1C, right). Interestingly, the binding of CENP-C and/or CENP-N to the CENP-A nucleosome induces further rotation, resulting in a canonically shaped nucleosome. It is possible that the forces at the CENP-A/CENP-A interface mediate nucleosome dynamics or stability, but more research is required to establish functional relevance of these observations.

### Nucleosome features contributed by DNA sequence

In organisms with a point centromere, like *S. cerevisiae*, the DNA sequence of the centromeric DNA is absolutely essential, as it is recognized by sequence-specific binding proteins. However, its importance in more complex eukaryotes is still controversial. In many organisms, natural centromeres are embedded in repetitive DNA, but at the same time, they can form and function elsewhere on the chromosome [5,52–54]. In humans, the centromeric, α-satellite, DNA, is a repetitive AT-rich sequence with the monomer length of 171 bp [3,4]. The 17-bp CENP-B box within this sequence is the only DNA sequence specifically recognized by the CCAN, and in particular CENP-B. However, since CENP-B knockout mice are viable [11,55] and neocentromeres [5,6] and human chromosome Y [56] are deprived of CENP-B, it seems like CENP-B function at the centromere is redundant.

Nevertheless, it is well established that the DNA sequence plays an important role in the formation and stability of the nucleosome itself [57,58]. Actually, the majority of the structural studies are done on nucleosomes wrapped with DNA sequences engineered with the aim to increase the nucleosome symmetry (palindromic sequences [59,60]) or nucleosome stability (super-positioning [61]). While engineered sequences make nucleosomes more amenable for biophysical studies, providing homogeneity and symmetry, the trade-off is the loss of valuable information about the nucleosome in its natural DNA environment. The research involving CENP-A nucleosomes is also affected by the need to artificially increase nucleosome stability in order to study them at high resolution. The crystal structure of the human CENP-A nucleosome was obtained using palindromic α-satellite DNA with CENP-B boxes at the ends (potentially increasing terminal DNA flexibility), while the use of the super-positioning 601 sequence [37,40], antibody fragments [40], cross-linking reagents [48] or detergents [44] were used to stabilize the CENP-A nucleosome for cryo-EM studies (Table 1). Interestingly, although the overall nucleosome shape is the same on different DNA sequences, a careful overlay points out clear deviations in the DNA path that is sequence specific. This indicates possible role of the DNA sequence in the centromere structure. While the tightly wrapped 601 sequence has the same path on both canonical and CENP-A nucleosome, with no or very slight deviations upon the binding of CENP-C or CENP-N, the natural centromeric α-satellite DNA deviates from that path in two places on the CENP-A nucleosome (Figure 1D). First, a 2-Å bulge between SHL1–SHL2 (Figure 1D, top) is present on all CENP-A structures with natural α-satellite DNA [40,48]. Interestingly, chromatin remodeling enzymes [62,63] and pioneering transcription factors [64,65] interact with the nucleosome at this site, while inducing nucleosome sliding or unwrapping. Other distinctive bulges of natural α-satellite DNA are detected between SHL 3.5–4.5 and −3.5 to −4.5, resulting in 2.9-Å widening of DNA gyres opposite from the dyad if CENP-A nucleosome on super-positioning 601 sequence (CENP-A^601^) and CENP-A nucleosome on natural α-satellite sequence (CENP-A^NAS^) are compared. However, in contrast with the SHL 1–2 location, which is basically insensitive to ligands binding to the nucleosome, this bulge decreases upon binding of the nucleosome-specific antibody and increases upon binding of CENP-C and CENP-N, resulting in total 4.2 Å gyre widening (Figure 1D, bottom). The binding of CENP-N accommodates a ∼1.5 Å shift of the DNA regions (SHL −3.5 and SHL 3.5) toward CENP-N binding site, which generates two symmetric CENP-N binding sites [48].

It is clear, ever since Lowary and Widom identified super-positioning 601 sequence for canonical nucleosome [58] that the DNA sequence affects the nucleosome stability. However, although computational methods are starting to address DNA sequence–nucleosome structure relationship [66] it is still extremely difficult to study experimentally at high resolution. Until recently, the majority of high-resolution nucleosome structures were obtained by X-ray crystallography, which imposes the constraints of crystal packing which, in turn, interferes with the high-quality crystal formation of nucleosomes wrapped with array of different sequences. Still, the impact of the DNA sequence on nucleosome structure was clearly observed in X-ray structures of nucleosome [57,67,68]. We hope the fast development of cryo-EM will enable a more thorough structural analysis of nucleosomes on different DNA sequences and advance our understanding of the DNA role in nucleosome structure. Present research has identified complex physico-chemical properties (like bendability and stretching) of a given DNA sequence (rather than the invariable order of nucleotides) to be responsible for DNA sequence stabilization of nucleosomes [57,69]. It is very possible that the CENP-A nucleosome has a particular and potentially slightly different preference for the DNA sequence in comparison with canonical nucleosome. This fine DNA sequence preference could be instrumental in positioning CENP-A nucleosome within monomers of centromere repeats. This in turn would lead to specific nucleosome phasing at the centromere, resulting in a unique centromeric chromatin structure. In fact, nucleosome phasing has been observed on human and mice α-satellite DNA monomers [39,70]. Nucleosomal DNA could also promote association of the CENP-A nucleosome with CCAN components and in that way assure stable centromeric chromatin. If indeed a specific DNA sequence emphasizes important dynamic features of the CENP-A nucleosome, one would expect a clear connection in the evolution of centromeric sequence and the CENP-A histone, but this study is still not available. Further understanding of the interplay between the DNA sequence and the histone octamer is necessary to resolve the role of the DNA sequence in centromere formation and functioning.

## CENP-A nucleosomes are specifically recognized by CENP-C and CENP-N

**Figure 2:**
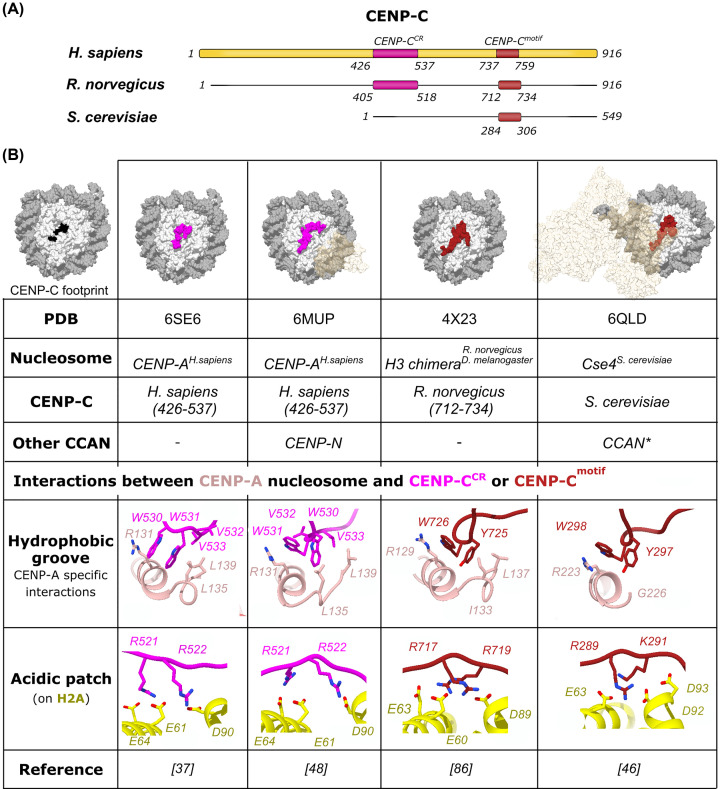
CENP-A nucleosome in complex with CENP-C (**A**) A schematic representation of CENP-C from *Homo sapiens, Rattus norvegicus* and *Saccharomyces cerevisiae*, indicating nucleosome-binding regions, CENP-C^CR^ and CENP-C^motif^, used in structural studies. (**B**) A table summarizing information revealed from published 3D-structures of nucleosomes in complex with CENP-C. A surface representation of the CENP-A nucleosome with mapped CENP-C footprint in black is shown on the far left. In the surface representation, histones are shown in light gray, DNA is colored dark gray, CENP-C^CR^ is magenta, the CENP-C^motif^ is dark red and other CCAN components shown in transparent beige. The CENP-C residues involved in the interaction with the acidic patch and the CENP-A C-terminal tail are shown as sticks.

**Figure 3:**
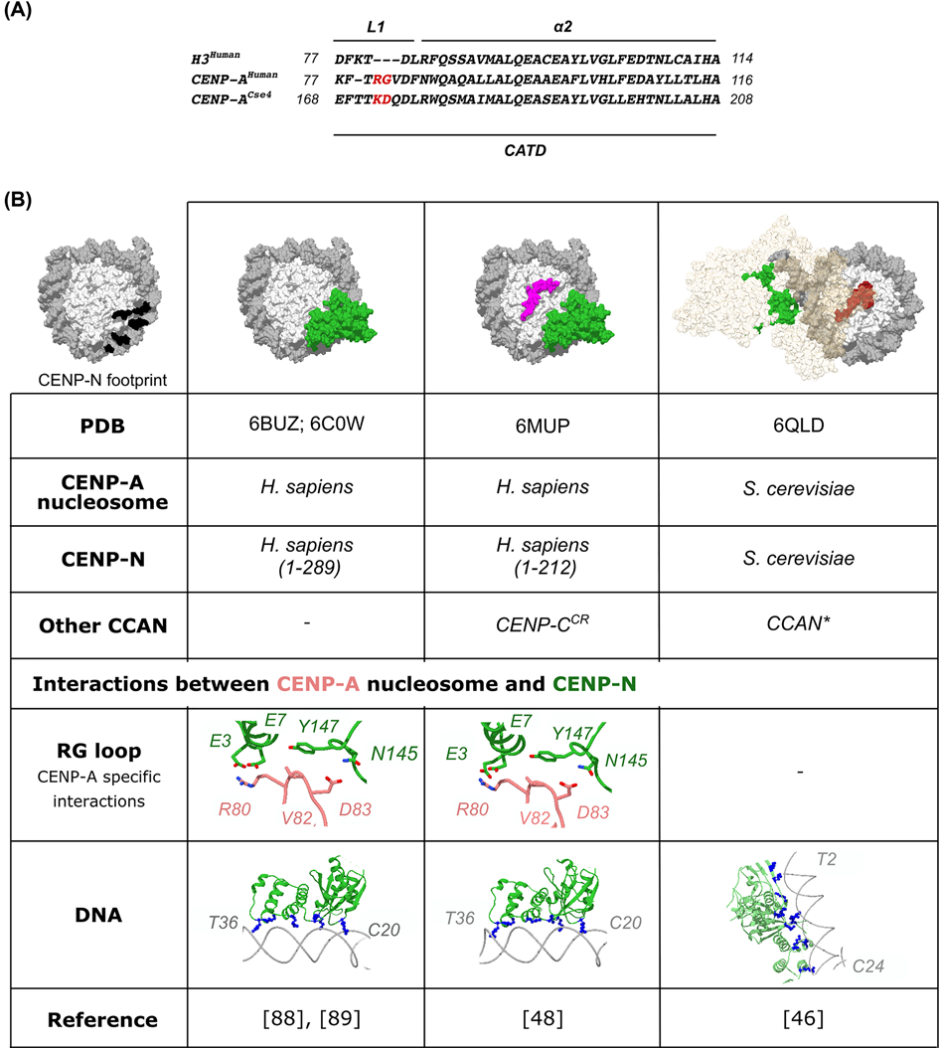
CENP-A nucleosome in complex with CENP-N (**A**) A multiple sequence alignment of the CATD region of CENP-A (*H. sapiens*) and CENP-A^Cse4^ (*S. cerevisiae*) with the corresponding region of H3 (*H. sapiens*). Insertions in the L1 loop are colored red. (**B**) A table summarizing information revealed from published 3D-structures of nucleosomes in complex with CENP-N. A surface representation of the CENP-A nucleosome with the mapped CENP-N footprint in black is shown on the far left. In the surface representation, histones are light gray, DNA is dark gray, CENP-N is green, CENP-C^CR^ is magenta, the CENP-C^motif^ is dark red, and the other CCAN components are shown in transparent beige. The CENP-N residues interacting with the CENP-A RG loop are shown as sticks. Positively charged residues (arginines and lysines), interacting with the nucleosomal DNA, are shown in blue.

The human CENP-A nucleosome is essential in establishing and maintaining a group of 16 proteins defining a centromere—the CCAN [71–73]. The complex is present at the chromosomes in all phases of the cell cycle, although it is proposed that the interaction between subunits changes through the cell cycle [50]. It is a platform for the formation of the kinetochore, which attaches the mitotic chromosome to the microtubules. The CCAN proteins can be divided into five subgroups based on biochemical and recruitment studies: CENP-C, CENP-L-N, CENP-H-I-K-M, CENP-T-W-S-X and CENP-O-P-Q-U-R [74–83]. CENP-C and CENP-N are the only two CCANs that bind directly to CENP-A nucleosomes [19,76,84–87]. The role of CENP-A in epigenetically marking the centromere directly depends on its ability to interact with CENP-C and CENP-N, but both interactions might vary through the cell cycle. Recently, the binding of both of these proteins to the CENP-A nucleosome has been elucidated at the atomic level [37,46,48,88–90], and findings have revealed that both CENP-C and CENP-N not only interact with CENP-A (specific interactions) but also make contacts elsewhere at the nucleosome (nonspecific interactions) (Figures 2 and 3). These studies have also revealed structural changes in the CENP-A nucleosome that are associated with the binding.

### CENP-A nucleosome in complex with CENP-C

CENP-C exists in many species across the animal, plant and fungi kingdom, but its sequence is evolutionally very dynamic [26,91,92]. It is a key CCAN component that plays a central role in the organization of the centromere and kinetochore recruitment [93]. Human CENP-C is a 934-amino acid long protein with many disordered regions that, besides binding to the CENP-A nucleosome, directly interacts with CENP-N-L, CENP-H-I-K-M and the DNA-binding protein, CENP-B [86,93–97]. The ability of CENP-C to bind several CCAN complexes, while at the same time directly connecting the CENP-A nucleosome through its C-terminus and the outer kinetochore components (KMN complex) through its N-terminus, together with having a dimerization domain that duplicates all of these interactions, have led to the proposal that CENP-C is a major organizer of the centromere [93]. The human CENP-C has two domains that specifically bind the CENP-A nucleosome, CENP-C central region 426–537 (CENP-C^CR^) and CENP-C motif 736–758 (CENP-C^motif^) (Figure 2A) [72,76,84,98]. The CENP-C^motif^ is one of the most evolutionarily conserved parts of the CENP-C protein [26,99], but it is neither necessary nor sufficient for kinetochore targeting in human cells [100]. On the other hand, CENP-C^CR^ is required for centromeric retention of the newly incorporated CENP-A [100]. Based on studies in chicken cells, the Fukagawa lab has proposed the interaction between the CENP-A nucleosome and CENP-C to be dependent on the cell cycle [49–51]. In the interphase cells, CENP-C is not interacting with the CENP-A nucleosome, while during mitosis CDK1-mediated phosphorylation of the Thr^651^ residue adjacent to CENP-C^motif^ (the only known CENP-C binding module in chicken cells), induces CENP-A nucleosome binding [49]. Until recently, the only available molecular insight into CENP-C binding was the X-ray crystal structure of a chimeric nucleosome bound with rat CENP-C^motif^ [86]. In the last 2 years, our understanding of the CENP-A nucleosome/CENP-C interactions was complemented with cryo-EM structures of human CENP-A nucleosomes, in complex with CENP-C^CR^ [37], and in complex with both CENP-C^CR^ and CENP-N [48], as well as a cryo-EM structure of *S. cerevisiae* CENP-A homolog (Cse4) in complex with 14 CCAN subunits, named Ctf19 complex in *S. cerevisiae* [46] (Cse4^Ctf19^). The CENP-A nucleosome has two binding sites for CENP-C, one on each face of the nucleosome, and makes a 1:2 complex with CENP-C. However, one copy of CENP-C is more disordered in almost all structures [37,46,48,86], which could be a consequence of instability of the complex during sample preparation (crystal formation or freezing in vitreous ice). The structural comparison reveals that CENP-C^CR^ and CENP-C^motif^ have similar modes of nucleosome binding, exploiting the hydrophobic interaction with the C-terminal tail of CENP-A, and electrostatic interaction with the acidic patch formed by H2A/H2B on the surface of the nucleosome (Figure 2B). The acidic patch is a hotspot for canonical nucleosome binding, and a similar interaction has been described for several chromatin complexes [101–104]. The CENP-A specific interaction is mediated by a hydrophobic stretch of amino acids that are longer in CENP-C^CR^, resulting in a stronger binding affinity toward the CENP-A nucleosome in comparison with CENP-C^motif^ [37]. Interestingly, despite evidence that CENP-C interacts with multiple modules within CCANs, those interactions are not visible in the Cse4^Ctf19^ complex, probably due to the dynamic nature of the protein parts involved [46]. Together, solution studies and high-resolution structures have indicated several structural changes that take place in the CENP-A nucleosome upon CENP-C binding. First, CENP-C^CR^ binding increases flexibility of the terminal DNA that is mediated through destabilization of the H2A C-terminal tail [37]. Interestingly, while the binding of the CENP-C^CR^ to CENP-A nucleosome destabilizes the DNA wrap, subsequent binding of CENP-N reverts this effect [40,48] (Figure 1C). Second, HDX, fluorescence and AFM studies detected rigidification and compaction of the CENP-A core upon CENP-C binding [85,105], and structural studies are in agreement with this [37,46,48]. Third, the CENP‐C binding rigidifies the N‐terminal tail of H4 in the conformation favoring H4^K20^ mono-methylation, required for the establishment and maintenance of functional centromeres [37,38,48]. Together, structural studies have started to reveal the effects and the complexity of the interaction between the essential CCAN protein, CENP-C, and the specialized centromeric nucleosome. However, a full understanding of how CENP-C organizes the centromere is awaiting more complete complexes.

### CENP-A nucleosome in complex with CENP-N

In humans, CENP-N is a 339-aa globular protein that binds the CENP-A nucleosome through its N-terminal region (1–286), and CENP-L through its C-terminal region (287-339). CENP-L in turn directly interacts with the CENP-H-I-K-M [93,96,100,106,107]. Although CENP-N directly binds the CENP-A nucleosome, its recruitment to the centromere is mediated by CENP-C during mitosis [89,96]. Initial biochemical studies have identified the exposed L1 loop within the CATD of CENP-A (Figure 3A) and the DNA, as binding determinants for CENP-N [76,84,100,108], and recent cryo-EM structures have confirmed the same binding elements [48,88–90].

The L1 loop is the only surface-exposed part of the CATD on the CENP-A nucleosome. It has two residues’ insertion, in comparison with H3—bulky charged Arg^80^ and small flexible Gly^81^ (usually referred as RG-loop). CENP-N engages with the RG-loop using both electrostatic and hydrophobic interactions (Figure 3B). The CENP-A^R80^ forms hydrogen bonds with both CENP-N^E3^ and CENP-N^E7^, while the CENP-N^Y147^ creates hydrophobic interaction with CENP-A^V82^. In comparison with the CENP-A nucleosome/CENP-C interaction, which has a relatively small footprint on the nucleosome (Figure 2B), the CENP-A nucleosome/CENP-N complex has a large interaction interface (2400 Å^2^), half of which is engaged in the CENP-N/DNA interaction (Figure 3B). However, the CENP-N/DNA interaction is not specific in nature, but rather several positively charged residues of CENP-N (Lys^15^, Arg^42^, Lys^45^, Lys^81^ and Arg^194^) interact with the backbone phosphates of DNA at the super-helical location SHL −2 to −3. The structure of the budding yeast CENP-N, in complex with the CENP-A (Cse4) nucleosome, which was solved in the presence of the other 13 Ctf19 components (Cse4^Ctf19^) [46], reveals striking differences from the structure of the human complex. Neither the interaction of CENP-N with L1 loop of CENP-A (Cse4) (^171^KD^172^ in CENP-A^Cse4^), nor the binding to the DNA at the SHL −2 to −3 are conserved in budding yeast. Instead, the CENP-N-L complex is reported to tightly bind the nucleosomal DNA, which is unwrapped from the nucleosome, using several positively charged Arginine and Lysine residues. However, no direct interaction is reported with the CENP-A (Cse4) nucleosome. Mutational analysis showed that, in the absence of CENP-C, the integrity of the CCAN complex depends on CENP-N-L/DNA interaction [46]. Pronounced differences in the mode of CENP-N binding to the CENP-A (Cse4) nucleosome, in comparison with the vertebrate CENP-A nucleosome, could reflect true differences between species or it could be a consequence of different *in vitro* sample preparation or a lack of other CCAN components in human complexes. Notably, the structures of Cse4^Ctf19^ and CENP-A/CENP-N [46,88–90] are asymmetric, with only one copy of the Ctf19 or CENP-N, respectively per nucleosome (two copies are expected based on solution studies), probably due to dissociation during freezing. Symmetrical occupancy of CENP-C and CENP-N, reported by Allu at al., was facilitated with mild cross-linking conditions (see Table 1) [48].

From the structure of the human CENP-A nucleosome, in complex with both CENP-C^CR^ and CENP-N [48], it is clear that these two modules can coexist on the same nucleosome without affecting each other’s binding. However, they both interact with the N-terminal tail of histone H4 and drive it in a specific orientation along the surface of the nucleosome, orienting it in a position that probably facilitates the centromere-specific H4^K20^ monomethylation essential for the epigenetic establishment of the kinetochore [109]. Furthermore, the concomitant binding of CENP-N and CENP-C^CR^ induces distinctive bulges of the DNA between SHLs 3.5/4.5 and SHLs −3.5/−4.5, which locally widen the DNA gyre distance by 3–4 Å (nucleosome gapping) accommodating a ∼1.5 Å DNA slide toward each of the CENP-N binding sites. This rearrangement is facilitated with natural α-satellite DNA, and it was not observed in the CENP-A nucleosome/CENP-N complexes on 601 DNA [48,88,89]. The same study reported two different stoichiometries of CENP-A/CENP-C/CENP-N complexes. One that has two copies of each, CENP-C and CENP-N, and the other one that has two copies of CENP-C and only one copy of CENP-N. The authors propose a model, based on cell experiments, where the chromosome condensation in mitosis results in the loss of one CENP-N molecule, generating asymmetrical arrangement at the CENP-A nucleosome that favors the formation of kinetochore.

In summary, the vertebrate CENP-N binds the RG-loop and the DNA between SHL2 and SHL3. The binding is facilitated on natural α-satellite DNA [48,100], where it further increases the sequence-specific DNA gyre gap opposite of the dyad. Also, the CENP-A nucleosome structures, in complex with CENP-C and CENP-N, have highlighted not only the importance of cumulative structural changes (for example, DNA unwrapping induced by CENP-C but stabilized again by CENP-N binding), but also the complexity of the stoichiometry within the CCAN that might be changing during the cell cycle [48,50,108].

## CENP-A nucleosome—a chromatin-embedded pedestal for the centromere

**Figure 4:**
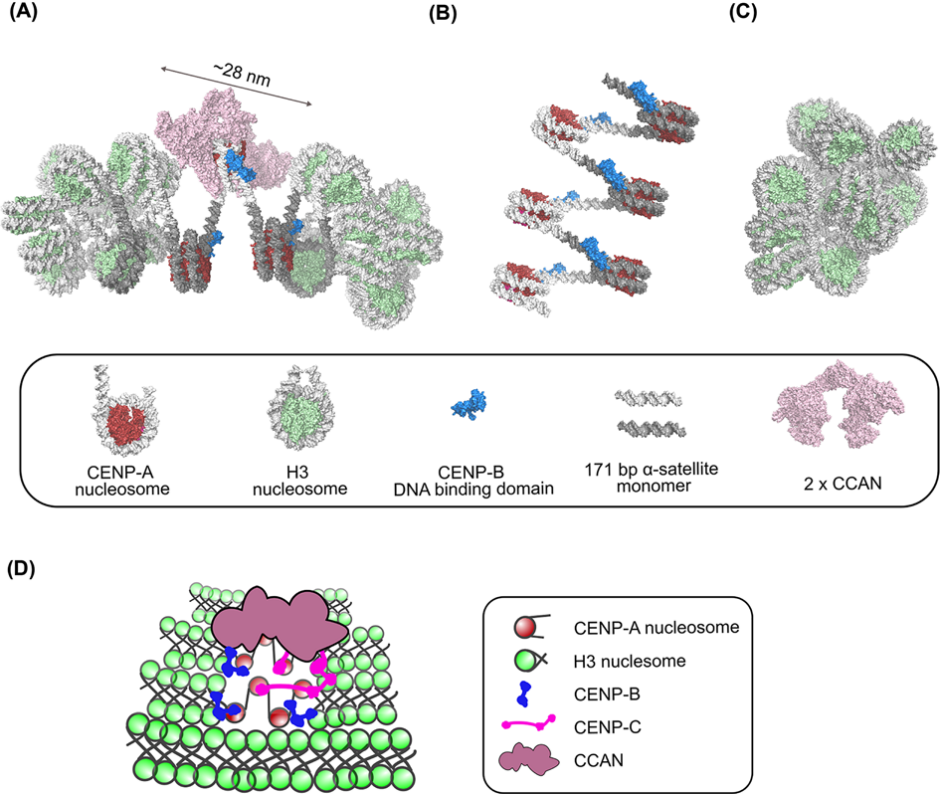
A model of the human CCAN complex in centromeric chromatin (**A**) 3D-model of the CCAN/centromeric chromatin complex. The nucleosome modeled harboring CCAN is built using structural information from the H3-CENP-A-H3 tri-nucleosome structure (6L49). CENP-C and CENP-N are modeled on the central nucleosome as in the human CENP-A/CENP-C/CENP-N structure (6MUP). The yeast CCAN complex structure (pink; extracted from 6QLD) deprived of the CENP-A^Cse4^ nucleosome, is docked on the central CENP-A nucleosome by aligning one copy of the CCAN complex on each CENP-N molecule. The DNA binding domain of CENP-B (DBD) (blue; 1HLV) is docked on a degenerated CENP-B box in the α-satellite sequence, as positioned in the CENP-A/CENP-C/CENP-N structure (6MUP). The DNA path was slightly adjusted to accommodate a kink observed in the CENP-B/DNA structure. The 171-bp monomers in the α-satellite sequence are colored light and dark gray (the nucleosome position is based on the CENP-A/CENP-C/CENP-N structure (6MUP)). It is obvious that nucleosomes adjacent to the CCAN loaded CENP-A cannot adopt a H3-H3-H3 packing (6L4A), so two CENP-A nucleosomes with open DNA ends are modelled on each side of the CENP-A, loaded with CCAN to avoid steric clashes. The H3 containing histone core is colored light green, and the CENP-A histone core is colored red. The size of the 2xCCAN:1CENP-A nucleosome complex is indicated. (**B**) A 3D-model of 6x CENP-A nucleosomes/CENP-B (DBD), based on the open DNA conformation of the CENP-A nucleosome in the H3-CENP-A-H3 tri-nucleosome structure (6L49). Notice the bigger spacing between nucleosomes. (**C**) A 3D-model of 18x H3 nucleosomes array built based on the H3-H3-H3 tri-nucleosome structure (6L4A). Notice the tight nucleosome packing imposed with DNA crossing over the nucleosome dyad. (**D**) A schematic diagram of human centromeric chromatin. H3 loaded chromatin is more compact than the CENP-A loaded chromatin. CENP-B binding is enforcing the CENP-A-like (looser) chromatin by introducing a kink close to the nucleosome entry/exit site. Because CENP-B has a dimerization domain, it is also bridging two nucleosomes. CENP-C is structuring the chromatin by binding four CENP-A nucleosomes (dimer with two nucleosome-binding regions) and thus increasing their local concentration. It also interacts with the CCAN.

The structure of the whole CCAN (Ctf19) complex from the budding yeast, in isolation [106] and in complex with the centromeric nucleosome [46], is a huge step toward understanding the molecular determinants of the centromere and kinetochore architecture. Although interactions between CCAN subunits and CENP-A nucleosome might be different in the more complex eukaryotes, the structure of the Cse^Ctf19^ gives a perception of the spatial organization around the CENP-A nucleosome, justifying the requirement for unwrapped DNA ends and accessible nucleosome faces that can accommodate CCAN binding. It is truly impressive to see a single nucleosome, ∼50 × 100 Å in size, supporting the megastructure of ∼200 × 300 Å (assuming 2 CCAN:1 nucleosome stoichiometry) (see model in Figure 4A). However, although this impressive structural work gives an idea of how the CCAN components are organized on the CENP-A (or Cse4) nucleosome, it, at the same time, opens other questions. How is this bulky structure embedded in chromatin? How does it re-organize with chromatin changes during the cell cycle, and how does it become available for kinetochore formation in mitosis?

CENP-A nucleosomes are present in the chromatin at fairly low levels. Indeed, according to Bodor at al. [110] even at the centromere, H3 nucleosomes are 25-times more abundant than the CENP-A nucleosomes; raising a question of how the CENP-A nucleosomes are organized within the centromere. Immunofluorescence and super-resolution microscopy studies [111–113] revealed that the CENP-A nucleosomes are interspersed between regions containing H3 nucleosomes, and cluster in G_1_. It is still not clear how the CENP-A nucleosomes organize in the higher order chromatin. We propose that distinct dynamic features of the CENP-A nucleosomes (flexible DNA ends and plastic histone core) generate chromatin with unique properties [36,43,108,114,115] which is more relaxed and can accommodate the bulky CCAN complex (Figure 4B). The tightly packed nucleosome arrays [36,108,114], where DNA crosses after it exits the nucleosome, are not compatible with the CCAN presence (Figure 4C). Instead, a flexible chromatin organization generated with untwisted linker DNA between nucleosomes [43] is needed in the neighborhood of the CCAN loaded CENP-A nucleosome (Figure 4A). Thus, the function of the vertebrate CENP-A nucleosome at the centromere might be dual. One fraction serves as a pedestal for the CCAN complex, while the other fraction participates in generating special chromatin environment, allowing space for the CENP-A^CCAN^ complex. In the point centromeres, like *S. cerevisiae*, the two functions are separated and both are genetically encoded [116,117]. The CENP-A (Cse4) assembled on 78–88 bp AT-rich CDEII is carrying Ctf19 complex, and surrounding CDEI and CDEIII DNA are binding Cfb1 and CBF3 complex, respectively, that are modifying surrounding chromatin.

Actually, recent efforts to visualize chromatin in cells using cryo-electron tomography (cryo-ET) and related techniques [118,119] are supporting a dynamic fluid-like organization of chromatin [120] rather than the rigid 30-nm chromatin fiber model based on *in vitro* chromatin studies that was widely accepted for decades. Comprehensive chromatin models based on an array of biophysical studies propose chromatin to be organized in distinct domains that have irregular fluid-like nucleosome organization [121]. Different cellular events like DNA replication, DNA repair/recombination and RNA transcription are all altering chromatin distribution between chromatin domains, and a phase separation is emerging as the mechanistic drive behind those processes [122]. Furthermore, the chromatin structure and nucleosome organization is dynamically changing during the cell cycle [119,123–125]. In the light of these observations, we propose that special dynamic properties of the chromatin loaded with CENP-A nucleosomes, distinguishes the centromere from the rest of the chromosome, but further studies are needed to confirm this model.

The intrinsic properties of the underlying DNA could also contribute toward the formation of the functional centromere. The repetitive nature of centromeric DNA could have a function in nucleosome spacing and positioning [39], and the DNA sequence properties are assuring the nucleosome ‘fit’ that promotes productive binding of CCAN components [48]. The distinct human CENP-A nucleosomes loaded with CCANs are further cross-linked by the CENP-C, a major organizer of the centromere, through its two nucleosome-binding sites and dimerization domain [37,93]. The DNA-binding CCAN components, CENP-N [76,84,88,89], CENP-T-W-S-X [81,126] and CENP-B [127] might help in further adjusting the centromeric chromatin structure and compaction. Intriguingly, CENP-B, the only CCAN component with DNA sequence-specific binding, has a redundant role in the established centromeric chromatin, but it becomes essential in *de novo* centromere formation on human artificial chromosomes [128]. One explanation could be that CENP-B conditions the chromatin for centromere formation, in the absence of pre-existing CENP-A, by binding close to the entry/exit site of the nucleosomes [129,130], which helps with nucleosome phasing [39,131] and DNA unwrapping (it induces a 59° kink [10]) and its dimerization domain [132] could play a role in specific chromatin cross-linking. Neocentromeres, which are natural centromeres that arise at the naïve chromatin (deprived from CENP-A and CENP-B) might exploit different mechanisms to establish functional centromere. A recent study by Kasinathan and Henikoff [133] used a bioinformatics approach to identify dyad symmetries in centromeres where CENP-B is absent and in neocentromeres, suggesting that DNA secondary structures could also play a role in centromere formation.

Finally, it is becoming more and more clear that strong epigenetic and weaker, but important, genetic traits both guide the centromere formation and function; the dynamic nature of which is yet to be understood.

## Conclusions and future perspectives

Amazing advances in the use of cryo-electron microscopy for single particle analysis cryo-ET and related techniques in the last decade [118,134,135] has revolutionized structural biology and introduced a much needed tool for studying complex molecular machines like the centromere and kinetochore. In the last 2 years, we have witnessed a large number of impressive high-resolution cryo-EM structures of the centromere and kinetochore complexes [37,46,48,88,89,106,136–138]. Yet, we are just starting to chip the tip of the iceberg when it comes to fully understanding these complex megastructures. Fortunately, other powerful and complementary techniques are developing, like structural mass-spectroscopy, computational modeling, chromosome capture techniques and super-resolution microscopy. An integrative approach, combining information from high-resolution structures with other complementary techniques that can assess fluctuations in dynamics, stoichiometry or precise location in the cell, will be a next step toward a holistic understanding of centromeres and kinetochores.

## Summary

- Centromeres are epigenetically specified by the histone H3 variant, CENP-A.
- CENP-A specific and DNA sequence-specific features are identified in high-resolution structures of the CENP-A nucleosome.
- CENP-N and CENP-C directly and specifically bind the CENP-A nucleosome and induce conformational changes.
- A specialized chromatin environment, contributed by both genetic and epigenetic elements, is defining the centromere.

## Abbreviations

aa: amino acid
AFM: atomic force microscopy
CATD: CENP-A targeting domain
CCAN: constitutive centromere associated network
cryo-EM: cryo-electron microscopy
cryo-ET: cryo-electron tomography
HDX: hydrogen-deuterium exchange
HFD: histone-fold domain
MNase: micrococcal nuclease
SAXS: small angle X-ray scattering
